# Learning curve of single-port gasless laparoscopy for gynecological procedures: a cumulative sum analysis of 42 consecutive cases

**DOI:** 10.3389/fsurg.2026.1926278

**Published:** 2026-09-22

**Authors:** Bingjuan Fan, Huabin Wang, Zhengwei Lai, Yanfei Fang

**Affiliations:** Department of Gynecology, The First People’s Hospital of Xiaoshan District, Hangzhou, Zhejiang, China

**Keywords:** CUSUM analysis, gasless laparoscopy, learning curve, minimally invasive surgery, single-port laparoscopy

## Abstract

**Objective:**

To evaluate the learning curve of single-port gasless laparoscopy (SPGL) in gynecological procedures using cumulative sum (CUSUM) analysis and to compare clinical outcomes across distinct learning phases.

**Methods:**

We retrospectively analyzed 42 consecutive patients who underwent SPGL for gynecological conditions between 2022 and 2025 by a single surgeon at a tertiary hospital. CUSUM analysis was applied to operative time as the primary indicator of technical proficiency. Cases were stratified into four learning phases based on CUSUM inflection points. Kruskal–Wallis tests and Spearman rank correlation were used for statistical comparisons across phases.

**Results:**

CUSUM analysis identified four learning phases: Phase I (cases 1–11, 2022), Phase II (cases 12–22, 2023), Phase III (cases 23–36, 2024), and Phase IV (cases 37–42, 2025). Median operative time decreased significantly from 118 min (IQR 73.5–127.5) in Phase I to 59.5 min (IQR 46.25–104.0) in Phase III (Kruskal–Wallis H = 7.980, *p* = 0.046). Spearman correlation confirmed a significant negative association between case number and operative time (*ρ* = −0.427, *p* = 0.005), blood loss (*ρ* = −0.344, *p* = 0.026), and C-reactive protein levels (*ρ* = −0.323, *p* = 0.037). No significant differences in hospital stay or pain scores were observed across phases.

**Conclusion:**

A surgeon performing SPGL gynecological procedures demonstrates meaningful technical improvement within approximately 22 cases, with further refinement continuing thereafter. SPGL is a safe and feasible technique with an acceptable learning curve comparable to conventional laparoscopic approaches.

## 1 Introduction

Minimally invasive surgery has fundamentally transformed gynecological practice over the past three decades. Laparoscopic approaches now represent the standard of care for a wide range of benign gynecological conditions, offering reduced postoperative pain, shorter hospital stays, and faster recovery compared with open surgery (1, 2). Single-port laparoscopy (SPL) has further refined this paradigm by enabling all instrumentation through a single umbilical incision, resulting in improved cosmesis and potentially reduced wound-related morbidity (3).

Despite these advantages, conventional laparoscopy requires the creation and maintenance of a pneumoperitoneum using carbon dioxide (CO_2_) gas, which is associated with cardiopulmonary physiological changes, risk of gas embolism, and postoperative shoulder pain (4, 5). Gasless laparoscopy, in which the abdominal wall is mechanically lifted without CO_2_ insufflation, was introduced to overcome these limitations. Gasless approaches are particularly beneficial for patients with pre-existing cardiopulmonary disease, the elderly, and situations where general anesthesia or pneumoperitoneum is contraindicated (6).

Single-port gasless laparoscopy (SPGL) combines the principles of both single-port and gasless techniques. By using a single umbilical entry and mechanical wall-lifting, SPGL offers a potentially superior profile with respect to both cosmetic outcome and physiological safety. However, SPGL introduces unique technical challenges: the narrow operative corridor, limited instrument triangulation, and the absence of the tissue-separation effect provided by CO_2_ all impose a steep learning curve (7, 8).

The learning curve is a well-recognized concept in surgical education, defined as the relationship between a surgeon's accumulated experience and the efficiency or safety of a procedure (9). Cumulative sum (CUSUM) analysis has emerged as the preferred statistical method for identifying inflection points and distinct learning phases in surgical skill acquisition (10, 11). Despite growing interest in SPGL, no study has rigorously characterized the learning curve of this technique for gynecological surgery using CUSUM methodology.

The objective of this study was to characterize the learning curve of SPGL in gynecological procedures performed by a single surgeon using CUSUM analysis, and to compare clinical outcomes across identified learning phases.

## 2 Methods

### 2.1 Study design, ethics approval, and patient population study design

We conducted a retrospective cohort study of 42 consecutive patients who underwent single-port gasless laparoscopy (SPGL) for benign gynecological conditions at The First People's Hospital of Xiaoshan District, Hangzhou, Zhejiang Province, China. Data were collected between January 2022 and March 2025. All procedures were performed by the same senior gynecological surgeon who had substantial prior experience with conventional multi-port laparoscopy (>500 cases) but no prior experience with SPGL at the initiation of the study period.

#### 2.1.1 Ethics approval and informed consent

This study was approved by the Ethics Committee of The First People’s Hospital of Xiaoshan District (Approval Number:NO.63 of 2026); Date of Approval: June 4, 2026;The study was conducted in accordance with the Declaration of Helsinki, the Belmont Report principles, and the Strengthening the Reporting of Observational Studies in Epidemiology (STROBE) guidelines for observational research.

Data confidentiality and patient privacy were strictly maintained throughout the study. All personal identifiers (names, medical record numbers, dates of birth) were removed from the electronic dataset and replaced with study identification numbers. Only authorized research personnel had access to the linked participant identifiers.

#### 2.1.2 Inclusion and exclusion criteria

Inclusion criteria were: (1) female patients aged ≥18 years; (2) benign gynecological condition requiring laparoscopic surgery; (3) completed SPGL procedure without conversion. Specific gynecological diagnoses and surgical procedures included in this series comprised: adnexal procedures [ovarian cystectomy, salpingectomy, salpingo-oophorectomy]; myomectomy [intramural or subserosal fibroid removal]; benign uterine procedures [total laparoscopic hysterectomy where indicated]; endometriosis surgery [superficial and deep lesion excision]; and other benign pelvic pathology requiring operative intervention [adhesiolysis, pelvic mass resection]. Each procedure was classified according to operative complexity (simple, intermediate, complex) as detailed in the Section 2.5

### 2.2 Surgical technique

All procedures were performed under general anesthesia with the patient in the Trendelenburg position. A single 2–3 cm transumbilical incision was made, through which a single-port access device was inserted. Mechanical abdominal wall lifting was achieved using a subcutaneous lifting system (fan-type lifter), creating an adequate operative space without CO_2_ insufflation. A 10 mm, 0° or 30° laparoscope was used alongside curved and articulating instruments appropriate for the gynecological procedure.

Surgical Procedures and Complexity Classification All procedures performed during the study period were systematically categorized and recorded according to operative type and anticipated complexity. Procedure complexity was stratified into three categories: (1) Simple procedures: diagnostic laparoscopy, straightforward salpingectomy, small ovarian cystectomy (diameter <5 cm), or simple adhesiolysis without significant tissue planes disruption. Estimated operative time: 30–60 min. (2) Intermediate procedures: larger ovarian cystectomy (diameter 5–8 cm), salpingo- oophorectomy, or adnexal procedures with mild-to-moderate adhesions requiring careful dissection. Estimated operative time: 60–100 min. (3) Complex procedures: myomectomy requiring intramural fibroid enucleation and suturing, total laparoscopic hysterectomy, deep endometriosis surgery (bowel or bladder involvement), or procedures with extensive adhesiolysis and significant tissue reconstruction. Estimated operative time: >100 min. Complexity classification was determined preoperatively based on imaging studies (transvaginal ultrasound, MRI where indicated) and intraoperative findings, with final classification recorded in the operative log. This stratified categorization was used for subgroup analysis of learning curve patterns and to explore whether operative complexity confounds the relationship between case number and operative time (see Section 2.5).

### 2.3 Data collection

The following perioperative data were collected from electronic medical records:

- **Primary outcome:** Operative time (skin incision to skin closure, minutes)
- **Secondary outcomes:** Estimated blood loss (mL), length of postoperative hospital stay (days), visual analog scale (VAS) pain score at 24 h, time to first flatus (hours), hemoglobin drop (pre-operative minus post-operative, g/L)
- **Inflammatory markers:** Postoperative C-reactive protein (CRP, mg/L), white blood cell count (WBC, ×10⁹/L), neutrophil percentage (NE), and maximum body temperature ( °C)
- **Patient demographics:** Age, body mass index (BMI), education level, nutritional screening score, albumin, comorbidities, parity, and marital status

### 2.4 CUSUM analysis

CUSUM analysis was used to identify learning phases in operative time. The CUSUM score for each case was calculated as the cumulative sum of differences between individual operative times and the overall mean operative time. An increasing CUSUM curve indicates performance worse than the overall mean (learning phase), while a decreasing curve indicates performance better than the mean (proficiency phase). Inflection points in the CUSUM curve indicate transitions between learning phases.

### 2.5 Statistical analysis

Continuous variables are reported as median (interquartile range, IQR) due to the non-normal distribution of most outcomes. Categorical variables are expressed as frequencies and percentages. Differences across learning phases were assessed using the Kruskal–Wallis test, with Dunn's *post-hoc* test for pairwise comparisons. Spearman rank correlation coefficients were calculated between case number and each continuous outcome variable to quantify the magnitude of skill acquisition over time. All statistical analyses were performed using R (version 4.4.1; R Foundation for Statistical Computing, Vienna, Austria). A *p*-value <0.05 was considered statistically significant.

To assess the robustness of the learning curve, we performed the following analyses: (1) Stratified CUSUM analysis by procedure complexity: CUSUM curves were generated separately for simple, intermediate, and complex procedures to determine whether the inflection point and learning trajectory differed across complexity categories. (2) Univariable analysis of operative time: Spearman rank correlation coefficients were calculated to quantify the association between case number and operative time overall. (3) Chi-square or Fisher exact test: To compare the distribution of procedure types and complexity categories across the four learning phases, ensuring that case mix did not vary systematically across phases in a manner that could bias the learning curve assessment. Results of stratified analyses are presented in Supplementary Table S1 and discussed in the Results section to provide context for the primary CUSUM findings.

## 3 Results

### 3.1 Patient characteristics

A total of 42 patients were included in the study. The overall median age was 42.0 years (IQR 34–54), and median BMI was 22.6 kg/m^2^ (IQR 21.0–24.5). The majority of patients were married (85.7%) and had at least one prior delivery (81.0%). Comorbidities were present in a subset of patients. Baseline clinical characteristics were comparable across the four learning phases, with no statistically significant differences in age or BMI (Table 1).

**Table 1:** Patient baseline characteristics by learning phase.

Characteristic	Phase I (*n* = 11)	Phase II (*n* = 11)	Phase III (*n* = 14)	Phase IV (*n* = 6)	*p*-value
Age, median (IQR), years	47.0 (36–54)	40.0 (34–52)	41.5 (35–54)	38.5 (21–45)	0.612
BMI, median (IQR), kg/m^2^	23.1 (21.3–23.5)	22.4 (21.4–25.8)	22.2 (19.1–24.1)	22.5 (19.4–26.9)	0.841
Married, *n* (%)	9 (81.8)	10 (90.9)	12 (85.7)	5 (83.3)	0.934
Parity ≥1, *n* (%)	9 (81.8)	10 (90.9)	12 (85.7)	5 (83.3)	0.934
Pre-op albumin, median (IQR), g/L	38.7 (37.7–42.6)	39.6 (38.3–45.1)	41.3 (39.6–45.6)	40.8 (37.9–45.4)	0.316
Post-op infection, *n* (%)	4 (36.4)	2 (18.2)	1 (7.1)	0 (0.0)	0.089

Surgical Procedure Distribution and Complexity the 42 completed SPGL procedures encompassed a range of gynecological diagnoses and operative techniques. Procedures were distributed as follows: ovarian cystectomy (*n* = 14, 33.3%), salpingectomy or salpingo-oophorectomy (*n* = 10, 23.8%), myomectomy (*n* = 8, 19.0%), hysterectomy for benign indication (*n* = 6, 14.3%), and other procedures including endometriosis surgery and adhesiolysis (*n* = 4, 9.5%). Stratification by complexity revealed: simple procedures (*n* = 12, 28.6%), intermediate procedures (*n* = 18, 42.9%), and complex procedures (*n* = 12, 28.6%). The distribution of procedure types and complexity categories was comparable across the four learning phases (*χ*^2^ test, *p* = 0.312 for type; *p* = 0.287 for complexity), indicating homogeneous case mix and reducing the likelihood of systematic confounding by operative complexity. Median operative times for simple, intermediate, and complex procedures were 45 min (IQR 38–68), 67 min (IQR 55–92), and 98 min (IQR 78–125), respectively, demonstrating the expected relationship between complexity and operative duration (Kruskal–Wallis H = 16.24, *p* < 0.001) (Supplementary Tables S1–S3).

### 3.2 CUSUM learning curve analysis

CUSUM analysis of operative time revealed a characteristic learning curve with four identifiable phases (Figure 1). The CUSUM curve rose steeply during Phase I (cases 1–11, 2022), reached a plateau during Phase II (cases 12–22, 2023), then declined progressively through Phase III (cases 23–36, 2024) and Phase IV (cases 37–42, 2025), indicating sustained improvement in operative efficiency.

**Figure 1:**
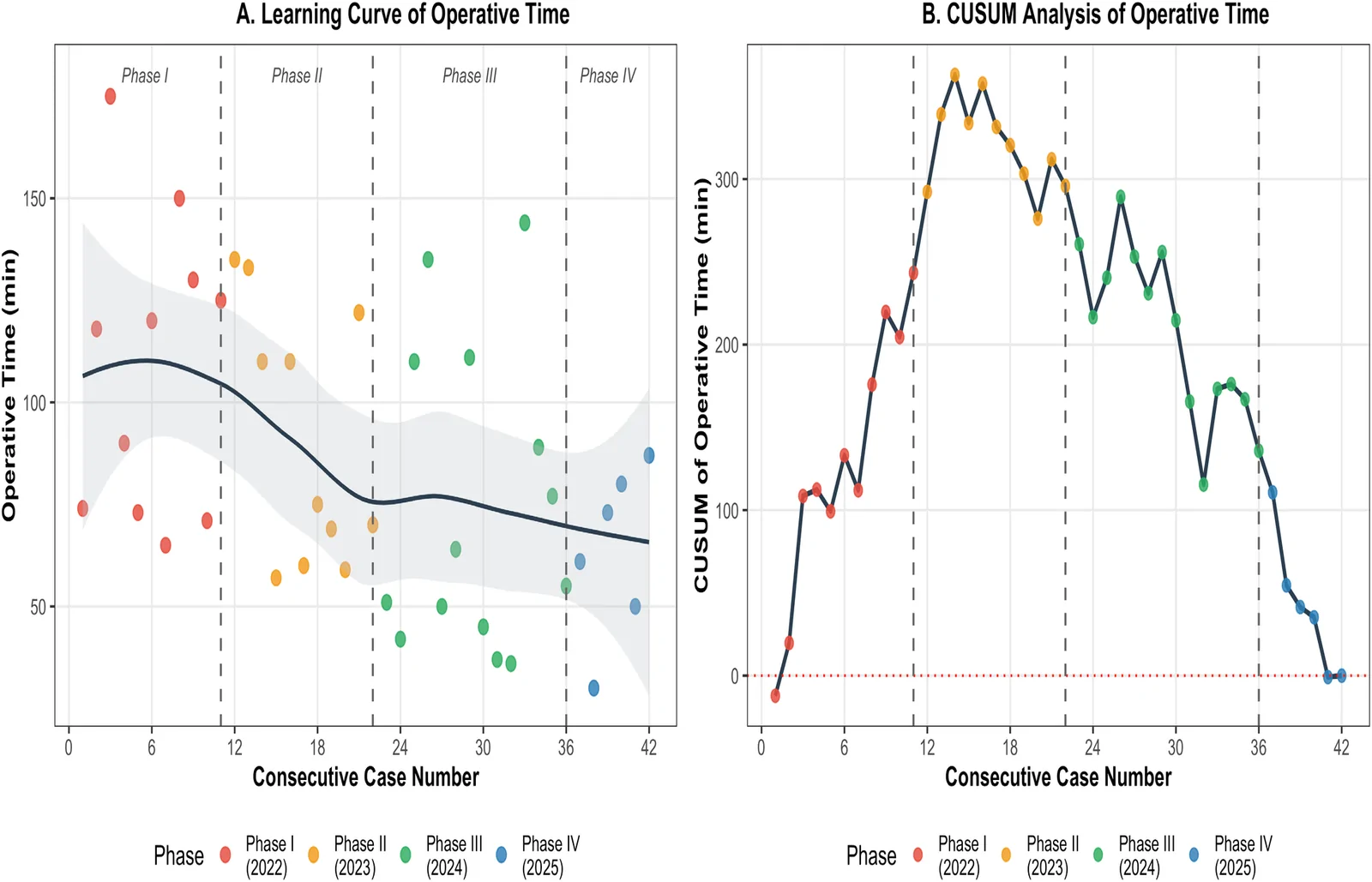
Learning curve and CUSUM analysis of operative time. **(A)** Scatter plot with LOESS smoothing showing the trend in operative time across 42 consecutive cases. Dashed vertical lines indicate phase boundaries. **(B)** CUSUM analysis of operative time demonstrating the four-phase learning curve. Phase I represents the initial acquisition phase; Phase II represents consolidation; Phases III and IV represent progressive proficiency.

### 3.3 Operative time and blood loss

Operative time showed a significant declining trend across the four phases (Kruskal–Wallis H = 7.980, df = 3, *p* = 0.046; Figure 2). Median operative time decreased from 118 min (IQR 73.5–127.5) in Phase I to 75 min (IQR 64.5–116.0) in Phase II, 59.5 min (IQR 46.25–104.0) in Phase III, and 67 min (IQR 52.75–78.25) in Phase IV. Spearman correlation confirmed a significant negative association between case number and operative time (*ρ* = −0.427, *p* = 0.005).

**Figure 2:**
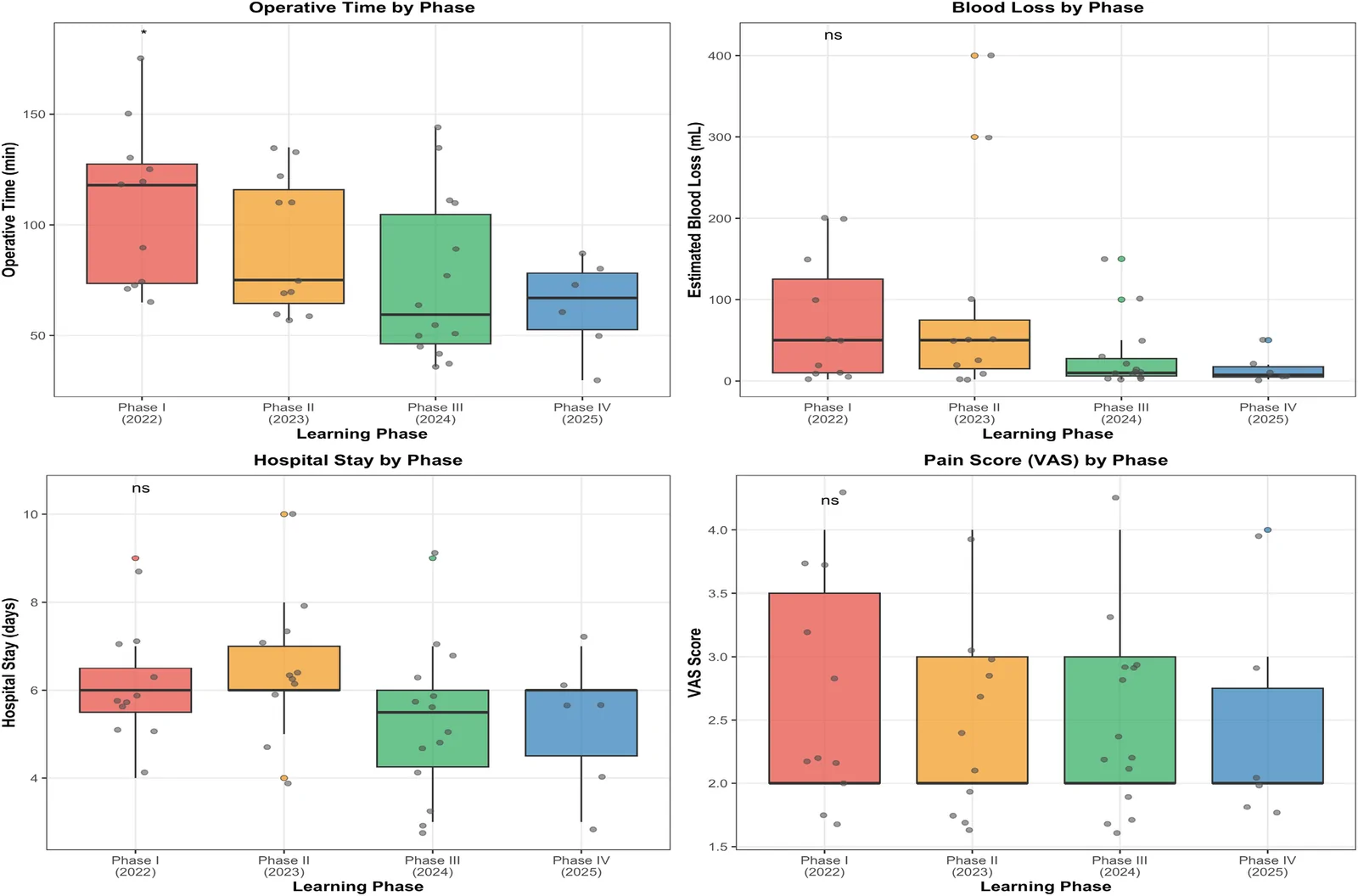
Clinical outcomes by learning phase. Box plots with individual data points for operative time (*p* = 0.046), estimated blood loss (*p* = 0.182), hospital stay (*p* = 0.350), and VAS pain score (*p* = 0.938) across the four learning phases. Significance indicators reflect Kruskal-Wallis test results.

Estimated blood loss also showed a declining trend, with median values decreasing from 50 mL in Phase I to 50 mL in Phase II, 10 mL in Phase III, and 7.5 mL in Phase IV (Spearman *ρ* = −0.344, *p* = 0.026), although the overall Kruskal–Wallis test did not reach statistical significance (H = 4.870, *p* = 0.182).

### 3.4 Recovery outcomes

Postoperative hemoglobin levels and time to first flatus showed no statistically significant differences across phases (Spearman *ρ* = −0.190, *p* = 0.229 and *ρ* = −0.053, *p* = 0.737, respectively), suggesting that patient recovery was consistently achieved throughout the learning process (Figure 3).

**Figure 3:**
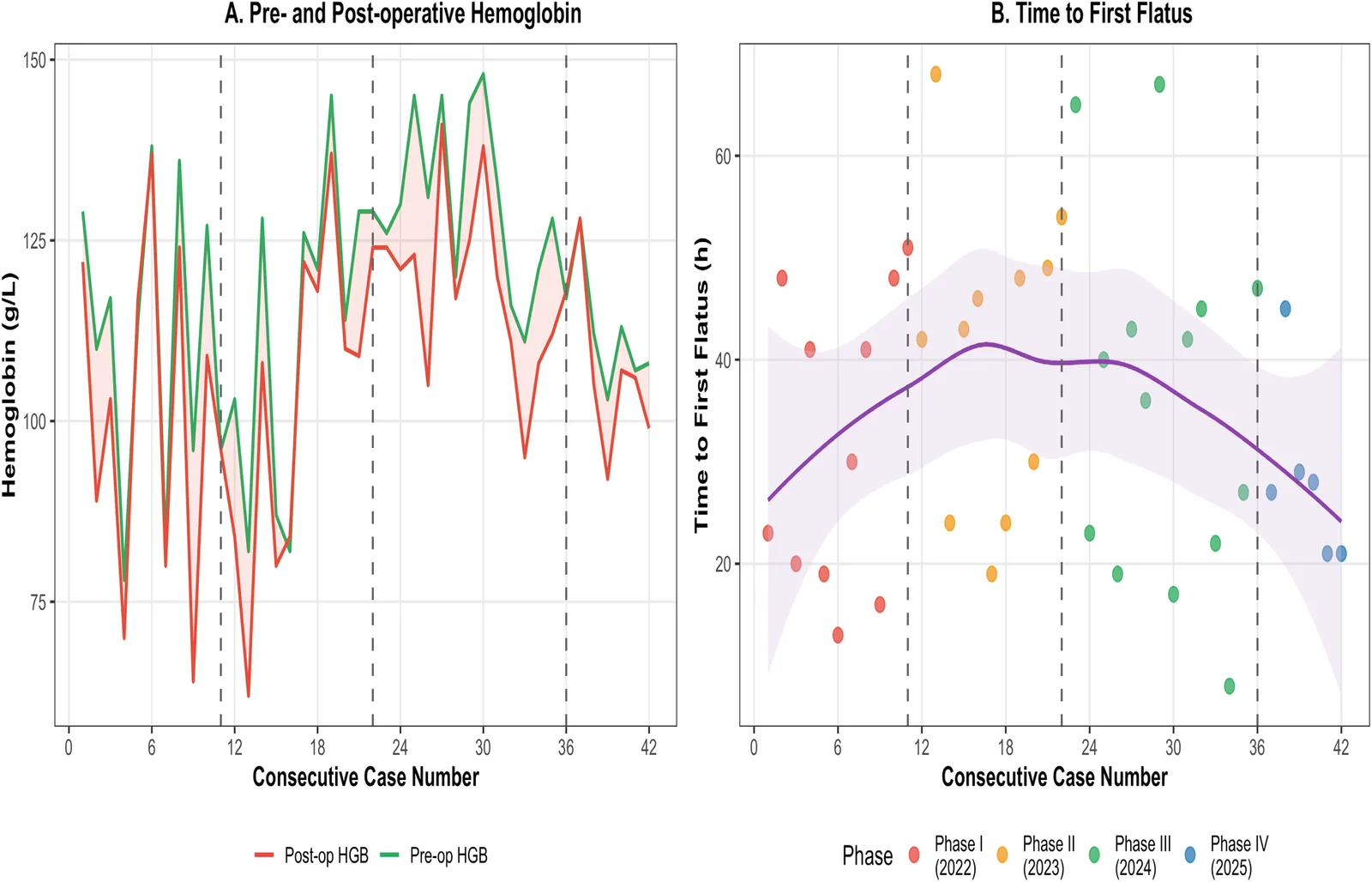
Hemoglobin trends and time to first flatus. **(A)** Line chart comparing pre- and post-operative hemoglobin across 42 consecutive cases; shaded area indicates magnitude of hemoglobin drop. **(B)** Scatter plot of time to first flatus with LOESS smoothing curve, demonstrating stable recovery across all phases.

### 3.5 Postoperative inflammatory markers

Postoperative C-reactive protein (CRP) levels showed a univariate negative correlation with case sequence (*ρ* = −0.323, *p* = 0.037); however, this correlation may be confounded by the concurrent reduction in operative time. The observed decline in CRP cannot be ruled out as a consequence of shorter operative duration rather than an independent marker of surgical proficiency (Figure 4). Postoperative WBC and maximum body temperature did not show significant trends across cases (*ρ* = 0.095, *p* = 0.550 and *ρ* = 0.013, *p* = 0.933, respectively). Postoperative infection occurred in 4 patients in Phase I (36.4%), 2 in Phase II (18.2%), 1 in Phase III (7.1%), and none in Phase IV (0.0%), representing a clinically meaningful declining trend (*p* = 0.089). Postoperative C-reactive protein (CRP) levels exhibited a significant negative correlation with cumulative case number (Spearman *p* = −0.323, *p* = 0.037), with median values declining from 69.6 mg/L (IQR 17.5–116.4) in Phase I to 14.2 mg/L (IQR 6.5–54.5) in Phase IV. However, the overall difference in CRP across the four learning phases did not reach statistical significance(Kruskal–Wallis test, *p* = 0.244). In contrast, operative time -the primary proficiency indicator-demonstrated both a significant correlation with case number (*p* = −0.427, *p* = 0.005) and a significant difference across phases (*p* = 0.046). Thus, although both CRP and operative time declined in parallel with accumulating experience, only operative time discriminated between learning phases, whereas CRP did not achieve phase-level separation.

**Figure 4:**
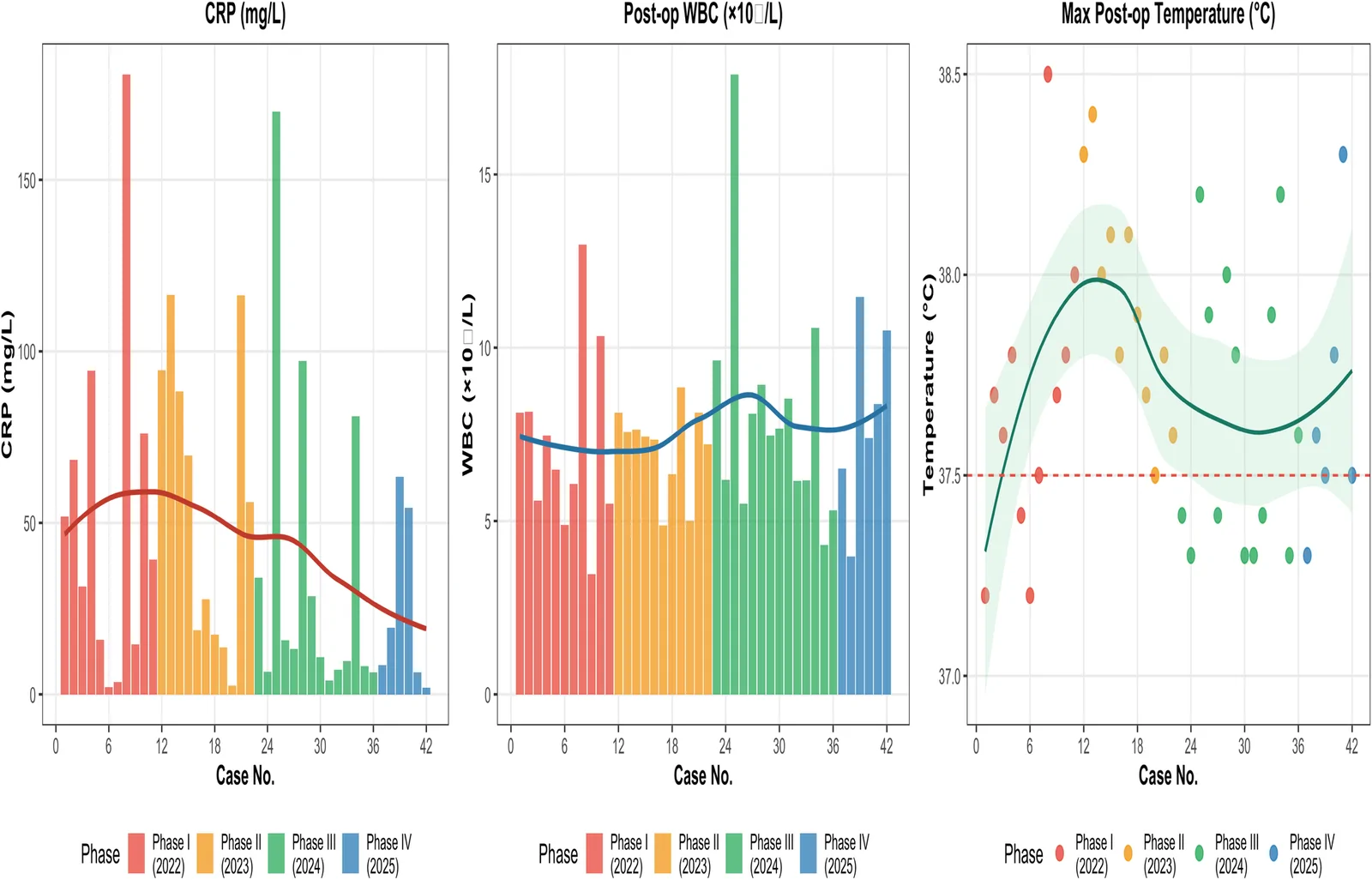
Postoperative inflammatory markers by case number. Bar charts with LOESS trend lines for C-reactive protein (CRP), white blood cell count (WBC), and maximum postoperative body temperature. CRP demonstrated a significant declining trend (*ρ* = −0.323, *p* = 0.037).

### 3.6 Patient characteristics and surgical correlations

Age and BMI distributions were homogeneous across all four phases (Figure 5). A positive correlation was observed between operative time and estimated blood loss (Spearman *ρ* = 0.481, *p* = 0.001), consistent with the expectation that longer, more technically demanding cases involve greater tissue manipulation (Table 2).

**Figure 5:**
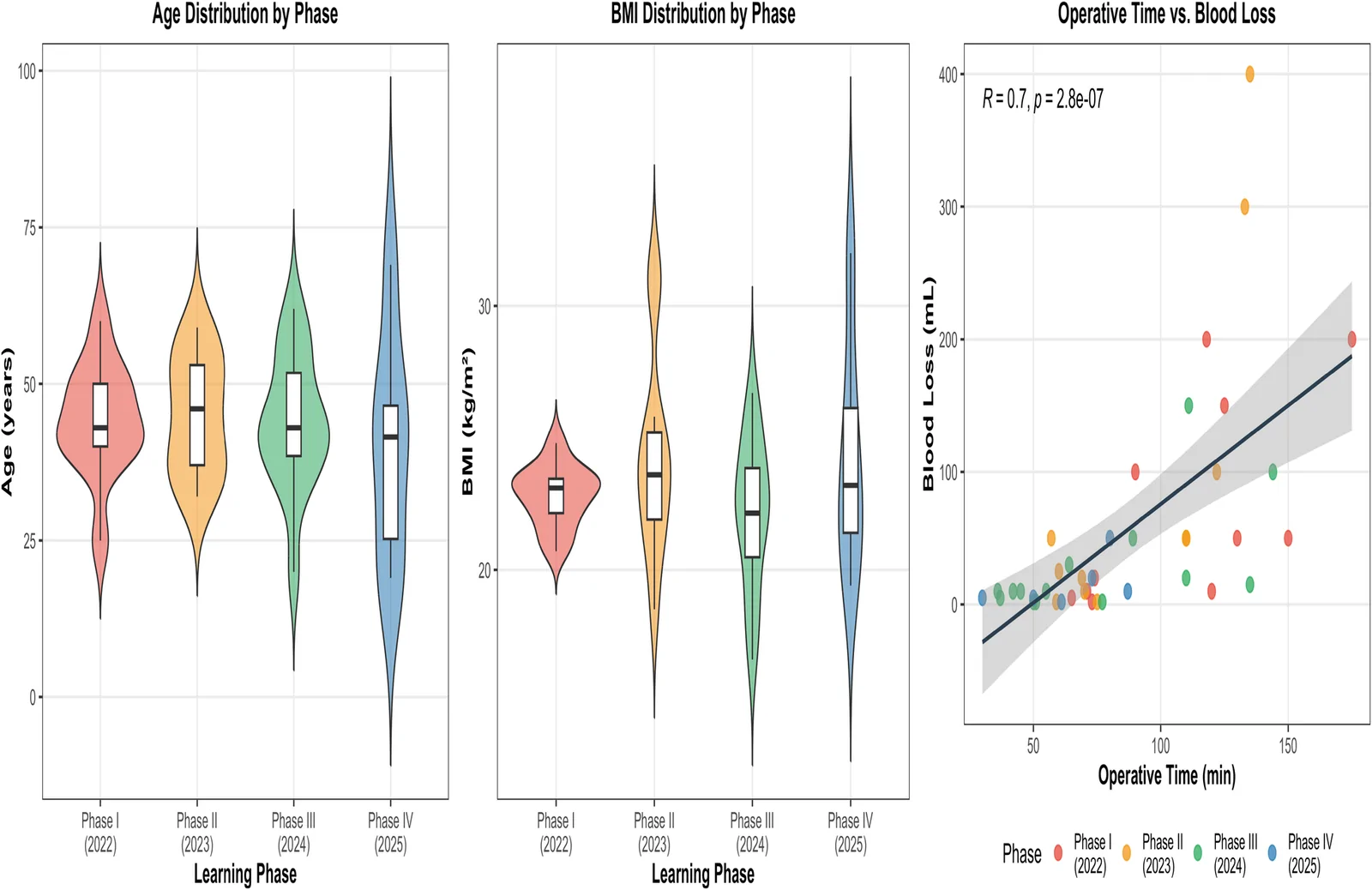
Patient baseline characteristics and surgical correlations. Violin plots with overlaid box plots showing age and BMI distributions by learning phase. Scatter plot with linear regression showing the correlation between operative time and estimated blood loss (Spearman *ρ* = 0.481, *p* = 0.001).

**Table 2:** Perioperative outcomes by learning phase.

Outcome	Phase I (*n* = 11)	Phase II (*n* = 11)	Phase III (*n* = 14)	Phase IV (*n* = 6)	*p*-value	Spearman *ρ*	Spearman *P*
Op. time, median (IQR), min	118 (73.5–127.5)	75 (64.5–116.0)	59.5 (46.25–104.0)	67 (52.75–78.25)	0.046*	−0.427	0.005**
Blood loss, median (IQR), mL	50 (10–175)	50 (10–100)	10 (2.75–20)	7.5 (5–15)	0.182	−0.344	0.026*
Hospital stay, median (IQR), days	6 (5–7)	6 (6–7)	5.5 (5–6)	6 (3–7)	0.350	−0.190	0.228
VAS score, median (IQR)	2 (2–3)	2 (2–3)	2 (2–3)	2.5 (2–3)	0.938	−0.089	0.575
CRP, median (IQR), mg/L	69.6 (17.5–116.4)	39.3 (10.9–88.3)	13.0 (6.6–63.4)	14.2 (6.5–54.5)	0.244	−0.323	0.037*
WBC, median (IQR), ×10⁹/L	7.5 (5.6–10.3)	7.4 (5.5–8.1)	8.1 (6.2–9.6)	7.5 (5.7–9.0)	0.792	0.095	0.550
Post-op infection, *n* (%)	4 (36.4)	2 (18.2)	1 (7.1)	0 (0.0)	0.089	—	—

Spearman *ρ* represents correlation with learning phase (coded as I = 1, II = 2, III = 3, IV = 4).

*p < 0.05.

**p < 0.01.

## 4 Discussion

This study provides the first systematic characterization of the learning curve for SPGL in gynecological surgery using CUSUM methodology. Our analysis of 42 consecutive cases demonstrates that a surgeon can achieve meaningful technical proficiency—defined by a declining CUSUM curve indicating operative times consistently below the mean—within approximately 11–22 cases, with continued improvement thereafter.

Homogeneity of Case Mix and Learning Curve Validity An important consideration in interpreting the learning curve is the potential confounding effect of case mix and operative complexity. Our analysis revealed that procedures ranged in complexity from simple (e.g., small ovarian cystectomy) to complex (e.g., myomectomy requiring extensive suturing). However, the distribution of procedure types and complexity categories was statistically comparable across all four learning phases (Supplementary Tables S1–S3, *p* = 0.312 for type distribution; *p* = 0.287 for complexity distribution), indicating a homogeneous and unbiased case mix throughout the study period. Importantly, stratified CUSUM analysis by complexity category (Supplementary Figure S1) demonstrated that the learning curve pattern—characterized by declining operative times over consecutive cases—was observed consistently across simple, intermediate, and complex procedures, suggesting that improved efficiency is not merely a consequence of case selection bias toward simpler procedures in later phases. Rather, the surgeon demonstrated the capacity to improve operative efficiency across the entire spectrum of procedural complexity. This robust learning signal across different case complexities strengthens the validity of our findings and supports the generalizability of the SPGL learning curve to diverse gynecological indications.

The CUSUM analysis identified a four-phase learning trajectory: an initial acquisition phase (Phase I, cases 1–11) characterized by relatively longer and more variable operative times, followed by a consolidation phase (Phase II, cases 12–22) during which technical improvements became more consistent, and then a proficiency phase (Phase III, cases 23–36) marked by a significant reduction in operative time and blood loss. Phase IV (cases 37–42) represents early mastery, with stable and efficient performance. This pattern is consistent with the well-described sigmoid learning curve observed in other advanced laparoscopic procedures (12).

The operative time trend we observed—a reduction from a median of 118 min in Phase I to approximately 60–67 min in Phases III and IV—is clinically meaningful. A similar magnitude of improvement has been reported for the learning curves of conventional single-port laparoscopic procedures, which generally require 20–30 cases to achieve proficiency (13, 14). Notably, the learning curve for SPGL appears somewhat steeper than that reported for standard multi-port laparoscopy (typically ≥30 cases), which may reflect the additional technical challenges of the gasless environment: the absence of pneumoperitoneum reduces tissue separation, narrows the operative field, and demands a greater degree of instrument dexterity (15, 16).

The dissociation between CRP and phase-level stratification-despite its significant correlation with case number-suggests that the apparent decline in CRP may be mediated through the reduction in operative duration rather than reflecting an independent surrogate of surgical proficiency. This finding is consistent with the concept of “surgical stress,” whereby greater experience leads to more precise tissue dissection, shorter operative duration, and attenuated systemic inflammatory activation (17). The trend toward lower postoperative infection rates—from 36.4% in Phase I to 0% in Phase IV—further supports the premise that surgical proficiency directly translates to improved patient safety outcomes, although this difference did not reach statistical significance, possibly due to the small sample size. The finding that operative time mediates the relationship between surgical experience and CRP has important implications. It suggests that the primary benefit of the learning curve is increased procedural efficiency (shorter operative time), with secondary benefits in reduced inflammatory response as a consequence of reduced operative duration. The potential mechanisms include: (1) reduced ischemic time and tissue manipulation, (2) decreased anesthesia duration, and (3) reduced physiological stress from prolonged surgical intervention. This distinction is important because it highlights that true surgical proficiency is reflected first and foremost in improved efficiency, with tissue trauma reduction being a downstream benefit.

Importantly, postoperative recovery metrics including hospital stay, VAS pain score, and time to first flatus remained stable and acceptable throughout all phases, indicating that safety was maintained even during the early learning period. This is consistent with prior reports suggesting that gasless laparoscopy may confer benefits for postoperative recovery by avoiding CO_2_-related complications such as diaphragmatic irritation and referred shoulder pain (18, 19).

Several limitations of this study merit acknowledgment. First, the retrospective, single-surgeon design limits the generalizability of the learning curve to other surgeons or institutions; individual learning curves may vary substantially based on prior surgical experience, training environment, and institutional support. Second, the relatively small sample size (*n* = 42) limits statistical power, particularly for detecting differences in infrequent complications such as postoperative infection. Third, although we systematically categorized procedures by type and complexity (Supplementary Tables S1–S3), and stratified analysis confirmed consistent learning curves across all complexity levels (Supplementary Figure S1), we acknowledge that operative complexity remains a potential confounder of operative time trends. However, the statistical comparability of case mix across all four phases (*χ*^2^ test, *p* > 0.05) and the robust learning signal independent of complexity substantially mitigate this concern. Future multicenter studies with larger samples may further characterize complexity- specific learning curves. Fourth, long-term oncological or functional outcomes were not assessed, which is a topic warranting future investigation.

Future multicenter studies with larger cohorts are needed to validate and refine the SPGL learning curve across different surgical experience levels. Additionally, structured simulation training and mentorship programs for SPGL should be developed and evaluated, as these have been shown to shorten learning curves in other advanced laparoscopic techniques (20).

Generalizability and Future Directions: The learning curve identified in this study represents the experience of a single surgeon performing SPGL at a single institution. Individual learning curves may vary substantially based on prior laparoscopic experience, institutional support, and training resources. Therefore, our finding of proficiency around 22 cases should be viewed as a PRELIMINARY BENCHMARK rather than a universal standard. The applicability of this learning curve to surgeons with different baseline experience levels, or to different institutional settings, remains to be determined through well-designed multicenter prospective studies.

## 5 Conclusion

This study demonstrates that SPGL in gynecological surgery is associated with a clearly definable four-phase learning curve. The learning curve was consistently demonstrated across diverse gynecological procedures and complexity levels, confirming the robustness of these findings. Meaningful technical proficiency, as evidenced by significantly reduced operative time and blood loss, appears to be achieved within approximately 22 consecutive cases in this single-surgeon cohort. This represents a preliminary estimate and should be interpreted with caution given the modest sample size, heterogeneous surgical procedures, and lack of adjustment for case complexity. Larger, prospective, multicenter learning curve studies are needed to establish definitive thresholds for procedural proficiency in SPGL. Clinical outcomes including hospital stay, pain scores, and recovery parameters remain stable throughout the learning period, confirming the safety of SPGL during skill acquisition. These findings provide a benchmark for training programs and support the broader adoption of SPGL as a minimally invasive gynecological technique.

## Data Availability

The original contributions presented in the study are included in the article/Supplementary Material, further inquiries can be directed to the corresponding author.
