# Supplementary material for: Learning curve of single-port gasless laparoscopy for gynecological procedures: a cumulative sum analysis of 42 consecutive cases
Source: Front Surg. 2026 Sep 22;13:1926278. doi: 10.3389/fsurg.2026.1926278 (PMC13640598; doi:10.3389/fsurg.2026.1926278)
Supplement: Supplementary file 1 [file Supplementaryfile1.docx]

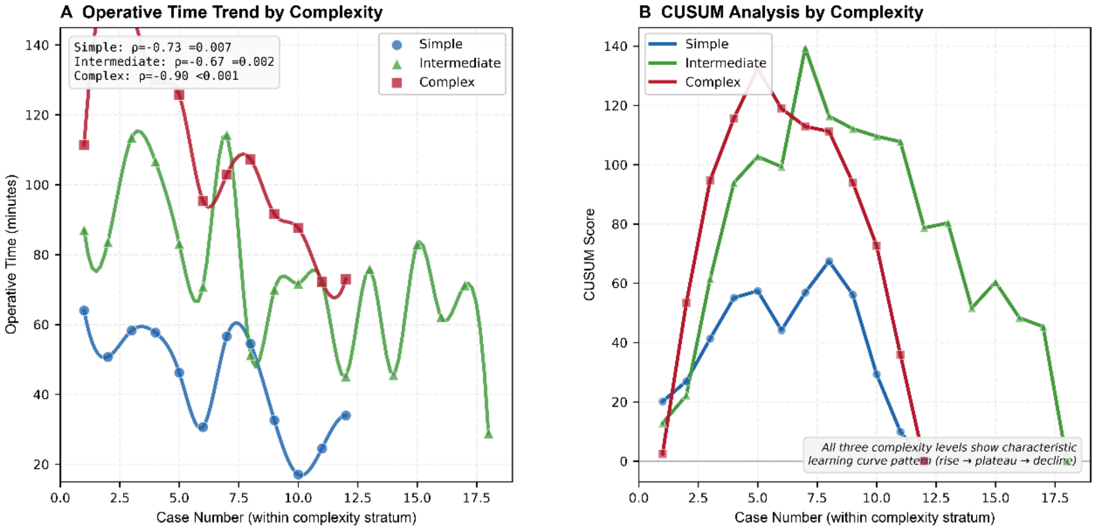
A Operative Time Trend by Complexity
Simple: ρ=-0.73 =0.007
Intermediate: ρ=-0.67 =0.002
Complex: ρ=-0.90 <0.001
Simple
Intermediate
Complex
Operative Time (minutes)
Case Number (within complexity stratum)
B CUSUM Analysis by Complexity
Simple
Intermediate
Complex
CUSUM Score
All three complexity levels show characteristic learning curve pattern (rise → plateau → decline)
Case Number (within complexity stratum)
