# Supplementary material for: Learning curve of single-port gasless laparoscopy for gynecological procedures: a cumulative sum analysis of 42 consecutive cases
Source: Front Surg. 2026 Sep 22;13:1926278. doi: 10.3389/fsurg.2026.1926278 (PMC13640598; doi:10.3389/fsurg.2026.1926278)
Supplement: Supplementary file 2 [file Supplementaryfile2.docx]

Supplementary Table 1

| **Table 1 Distribution of Surgical Procedures Across Learning Phases** | | | | | |
| --- | --- | --- | --- | --- | --- |
| **Procedure** | **Phase I (n=11)** | **Phase II (n=11)** | **Phase III (n=14)** | **Phase IV (n=6)** | **Total n (%)** |
| Ovarian cystectomy | 4 (36.4) | 3 (27.3) | 5 (35.7) | 2 (33.3) | 14 (33.3) |
| Salpingectomy/SO | 3 (27.3) | 3 (27.3) | 3 (21.4) | 1 (16.7) | 10 (23.8) |
| Myomectomy | 2 (18.2) | 2 (18.2) | 3 (21.4) | 1 (16.7) | 8 (19.0) |
| Hysterectomy | 1 (9.1) | 2 (18.2) | 2 (14.3) | 1 (16.7) | 6 (14.3) |
| Other* | 1 (9.1) | 1 (9.1) | 1 (7.1) | 1 (16.7) | 4 (9.5) |
| **χ² value** |  |  |  |  | **4.82** |
| **P value** |  |  |  |  | **0.312** |

Supplementary Table 2

| **Table 2 Procedure Complexity and Operative Duration** | | | |
| --- | --- | --- | --- |
| **Complexity** | **n (%)** | **Median (min)** | **IQR (min)** |
| Simple | 12 (28.6) | 45 | 38–68 |
| Intermediate | 18 (42.9) | 67 | 55–92 |
| Complex | 12 (28.6) | 98 | 78–125 |
| ***Note:*** *Kruskal-Wallis H test; IQR, interquartile range.* ***H = 16.24, P < 0.001.*** | | | |

Supplementary Table 3

| **Table 3 Distribution of Procedure Complexity Across Learning Phases** | | | | | |
| --- | --- | --- | --- | --- | --- |
| **Complexity** | **Phase I (n=11)** | **Phase II (n=11)** | **Phase III (n=14)** | **Phase IV (n=6)** | **Total n (%)** |
| Simple | 3 (27.3) | 3 (27.3) | 4 (28.6) | 2 (33.3) | 12 (28.6) |
| Intermediate | 5 (45.5) | 5 (45.5) | 6 (42.9) | 2 (33.3) | 18 (42.9) |
| Complex | 3 (27.3) | 3 (27.3) | 4 (28.6) | 2 (33.3) | 12 (28.6) |
| **ς² value** |  |  |  |  | **2.49** |
| **P value** |  |  |  |  | **0.287** |

** Other procedures include endometriosis surgery and adhesiolysis.*

*Abbreviations: SO, salpingo-oophorectomy; IQR, interquartile range; SPGL, single-port gasless laparoscopy.*

*χ² test was used to compare distributions across learning phases.*
